# Posterior bladder-associated pelvic cystic lesion with histologic features consistent with mesenteric cyst in an infant with a functionally solitary kidney: a diagnostic uncertainty case report

**DOI:** 10.3389/fped.2026.1830291

**Published:** 2026-06-19

**Authors:** ZainEdeen Zyadah, Joyce Morcos, Rami Muallem, Aya Shaabneh

**Affiliations:** Faculty of Medicine, Al-Quds University, Jerusalem, Palestine

**Keywords:** cryptorchidism, hydronephrosis, laparoscopic marsupialization, mesenteric cyst, solitary kidney

## Abstract

**Background:**

Posterior bladder-associated pelvic cystic lesions in male infants are diagnostically challenging, particularly when congenital anomalies of the kidney and urinary tract coexist. Deep pelvic cystic lesions may mimic mesenteric cyst, bladder duplication, bladder diverticulum, ectopic ureter-related pathology, seminal vesicle cyst, or Zinner-spectrum malformation.

**Case presentation:**

We report a male infant born at 39 weeks' gestation with severe right kidney atrophy, a functionally solitary left kidney with hydroureteronephrosis, a posterior bladder-associated pelvic cystic lesion, and bilateral undescended testes. Initial micturating cystourethrogram showed no vesicoureteral reflux, but posterior urethral valves could not be definitively excluded. No preoperative or intraoperative cystourethroscopy was performed. During follow-up, he had a culture-positive suspected urinary infection with extended-spectrum beta-lactamase-producing *Klebsiella pneumoniae*; urine collection method, colony count, and complete pyuria documentation were unavailable. Dimercaptosuccinic acid scanning showed marked functional asymmetry, with the left kidney contributing 96% and the right kidney 4%. Laparoscopic exploration revealed a large pelvic cystic lesion densely adherent to the posterior bladder wall and apparently fused with it, with both testes attached posteriorly. Complete excision was considered unsafe; therefore, marsupialization and bilateral orchiopexy were performed. Histopathological examination of a limited cyst-wall specimen showed benign cystic tissue with features interpreted as consistent with mesenteric cyst, without immunohistochemistry or detailed epithelial characterization.

**Discussion:**

After accidental drain removal, urine passage from the abdominal tract was reported, and ultrasound later demonstrated a supravesical tract. Postoperative cystoscopy reportedly excluded posterior urethral valves, bladder duplication, bladder diverticulum, ectopic ureteric opening, and internal bladder fistula, but the detailed report, intraoperative media, pathology images, and additional postoperative tract imaging were unavailable.

**Conclusion:**

This case is best interpreted as a posterior bladder-associated pelvic cystic lesion with histologic features consistent with mesenteric cyst, rather than a definitively proven mesenteric cyst. It highlights the need for cystourethroscopic evaluation, broad genitourinary differential diagnosis, clinicopathological correlation, and transparent reporting of diagnostic uncertainty.

## 1 Introduction

Mesenteric cysts are uncommon benign intra-abdominal lesions arising from mesenteric tissue. In children, they are usually described as abdominal lesions and may present with abdominal distension, pain, palpable mass, vomiting, intestinal obstruction, or may be discovered incidentally (1–5). Imaging is required to localize the lesion, define its relationship to adjacent structures, and guide operative planning (1, 4, 5). Ultrasonography and computed tomography are commonly used in the evaluation of pediatric intra-abdominal cystic lesions (1, 5). However, imaging may not always establish the exact origin of a pelvic cyst, particularly when the lesion lies adjacent to the urinary bladder. In male infants, posterior bladder-associated pelvic cystic lesions require a broad differential diagnosis, including mesenteric cyst, bladder duplication, bladder diverticulum, ectopic ureter-related cystic lesions, seminal vesicle cyst, and Zinner-spectrum malformation (6–8).

Complete excision is generally considered the preferred treatment for mesenteric cysts when technically feasible, because incomplete treatment may carry risks of recurrence, persistent drainage, or need for reoperation (1, 3, 4). However, complete excision may be unsafe when the cyst is densely adherent to critical structures such as the bladder, ureters, gonadal vessels, or bowel. In children with a congenital functionally solitary kidney or markedly asymmetric kidney function, diagnostic and therapeutic decisions carry additional risk. These patients require long-term surveillance for kidney function decline, hypertension, proteinuria, and associated urinary tract anomalies (9, 10).

This report describes a posterior bladder-associated pelvic cystic lesion in an infant with severe right kidney abnormality, a functionally solitary left kidney, hydroureteronephrosis, and bilateral undescended testes. A limited cyst-wall specimen showed histologic features interpreted as consistent with mesenteric cyst, while the lesion's posterior bladder location, close relationship to the posterior bladder wall, suspected postoperative urinary drainage tract, and limited source documentation left its full anatomical origin uncertain.

## 2 Case presentation

A male infant, the first child of second-degree cousin parents, was born on 24 January 2025 at 39 weeks' gestation by uncomplicated vaginal delivery. Birth weight was 2.8 kg. No antenatal abnormalities were reported. The father had type 1 diabetes mellitus, and there was no known family history of congenital kidney disease or inherited genetic disease. Early postnatal imaging demonstrated severe right kidney atrophy, left hydroureteronephrosis, a cystic pelvic structure posterior to the urinary bladder, and bilateral undescended testes. On the first day of life, the infant was clinically well, active, not dehydrated, and not in respiratory distress. Abdominal examination was soft and lax, without palpable mass or tenderness. Initial laboratory testing showed serum creatinine 1.0 mg/dL and blood urea nitrogen 12.5 mg/dL. Blood gas analysis showed pH 7.42, PaCO₂ 27.8 mmHg, HCO₃⁻ 20.3 mmol/L, base excess −4.7 mmol/L, and lactate 3 mmol/L. The infant was transferred for neonatal monitoring, urine-output surveillance, and further investigation.

A micturating cystourethrogram performed on 28 January 2025 demonstrated no vesicoureteral reflux, but the available report stated that posterior urethral valves could not be definitively excluded. A Foley catheter was maintained with a plan for reassessment. Later clinical documentation described the study as normal, but the complete repeat report and VCUG images were unavailable for independent verification. Preoperative or intraoperative diagnostic cystourethroscopy was not performed. At the time, the lesion was approached as a persistent extravesical pelvic cystic mass, and the operative plan proceeded as diagnostic laparoscopic exploration. Because the initial VCUG could not definitively exclude posterior urethral valves, and because the lesion was posterior to the bladder in a male infant with severe ipsilateral kidney abnormality, cystourethroscopy could have provided additional anatomical assessment of the posterior urethra, ureteric orifices, bladder mucosa, possible diverticular neck, ectopic ureteric insertion, internal fistulous opening, or bladder duplication.

At 26 days of age, the infant was admitted after a positive urinalysis during routine follow-up. He had no fever, vomiting, poor feeding, cyanosis, abnormal movements, altered consciousness, or bowel changes, and examination remained unremarkable apart from bilateral undescended testes. Urine culture grew extended-spectrum beta-lactamase-producing *Klebsiella pneumoniae*, while blood cultures showed no growth. The urine collection method, colony count, and complete pyuria documentation were unavailable. Because strict pediatric urinary tract infection diagnosis requires compatible clinical findings, pyuria, and a positive urine culture obtained by an appropriate method, this episode is reported as a culture-positive suspected urinary infection rather than a definitive urinary tract infection according to strict pediatric criteria (11). Empirical cefotaxime was initiated and later escalated to meropenem according to antimicrobial susceptibility, with clinical improvement and a negative control urine culture. Kidney function was monitored serially because of the functionally solitary left kidney. Serum creatinine was 1.0 mg/dL on the first day of life, 0.28 mg/dL during the February admission, 0.33 mg/dL perioperatively in May, and 0.96 mg/dL during the November admission. For context, Colantonio et al. reported a serum creatinine reference interval of approximately 0.31–0.53 mg/dL for male infants aged 15 days to less than 1 year using the Jaffe method; therefore, elevated values in this case are reported descriptively rather than labeled as preserved kidney function (12).

Serial ultrasound examinations confirmed severe right kidney atrophy and left hydroureteronephrosis. Abdominal ultrasound on 17 March 2025 demonstrated a well-defined anechoic structure, shaped like an underfilled urinary bladder, located posterior to the urinary bladder and measuring approximately 5 × 2 cm. The lesion was considered possibly mesenteric in origin, but further investigation was recommended. The right kidney was not visualized, while the left kidney demonstrated moderate hydronephrosis, with kidney pelvic diameter of approximately 1 cm. A dimercaptosuccinic acid scan on 28 March 2025 demonstrated marked functional asymmetry, with the left kidney contributing 96% and the right kidney 4%, consistent with a functionally solitary left kidney. Contrast-enhanced CT on 7 May 2025 demonstrated a well-circumscribed, thin-walled, homogeneous fluid-attenuation cystic lesion located posterosuperior to the urinary bladder and closely related to the posterior bladder wall, without visible septations, mural nodularity, calcification, or solid enhancing component (Figure 1). The right kidney was not visualized or was severely atrophic, and the left kidney demonstrated compensatory prominence with pelvicalyceal dilatation/hydronephrosis (Figure 2).

**Figure 1:**
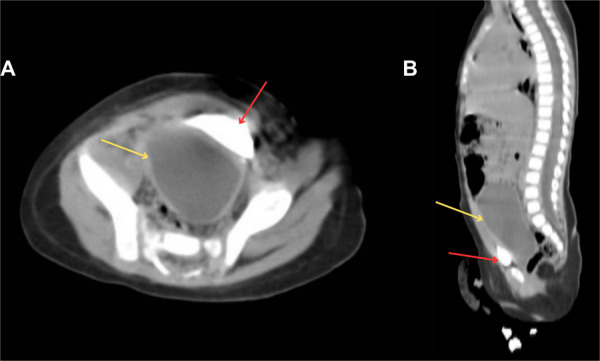
Contrast-enhanced CT abdomen and pelvis demonstrating a posterior bladder-associated pelvic cystic lesion. **(A)** Axial image showing a well-circumscribed, homogeneous fluid-attenuation cystic lesion located posterior to the urinary bladder and closely related to the posterior bladder wall. The yellow arrow indicates the cystic lesion, and the red arrow indicates the contrast-filled urinary bladder. **(B)** Sagittal reconstructed image demonstrates the craniocaudal extent of the lesion and its close anatomical relationship to the posterior bladder wall. CT demonstrated the posterior bladder-associated location of the lesion but did not definitively establish its embryologic or anatomical origin.

**Figure 2:**
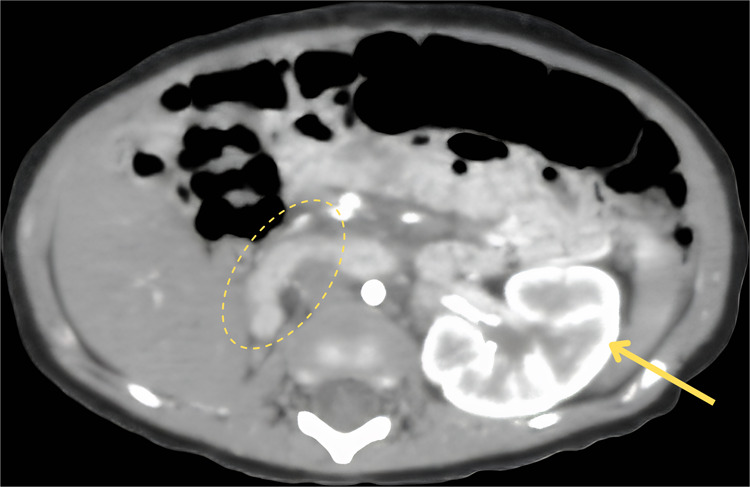
Contrast-enhanced CT abdomen and pelvis demonstrating severe right kidney abnormality and compensatory prominence of the functionally solitary left kidney. The yellow dashed oval indicates the region of the severely atrophic right kidney or right kidney fossa, and the yellow arrow indicates the left kidney. The left kidney demonstrated compensatory prominence with associated pelvicalyceal dilatation/hydronephrosis. These findings supported cautious diagnostic and operative planning because of limited kidney functional reserve.

Given the persistent posterior bladder-associated pelvic cystic lesion, hydroureteronephrosis, functionally solitary kidney, culture-positive suspected urinary infection, and concern for either a mesenteric cyst or bladder-associated anomaly, the multidisciplinary team proceeded with diagnostic surgical exploration. The delay from initial neonatal detection to surgery reflected staged neonatal stabilization, antimicrobial treatment, serial imaging, functional kidney assessment, CT characterization, diagnostic uncertainty, family-related delay, and operative scheduling constraints within a resource-limited public healthcare system.

On 20 May 2025, laparoscopic exploration was performed under general anesthesia. The operative record documented placement of a 5-mm umbilical trocar and two additional 5-mm working ports. Intraoperatively, the surgeons encountered a large posterior bladder-associated pelvic cystic lesion densely adherent to the posterior bladder wall and apparently fused with it. Both testes were attached to the posterior aspect of the lesion. One operative note used the phrase “communicating with bladder”; however, no intraoperative cystourethroscopy, cystography, methylene blue test, or direct endoluminal confirmation was performed. In the absence of endoluminal confirmation, the intraoperative finding was interpreted as dense adherence or apparent fusion to the posterior bladder wall rather than a proven pre-existing epithelialized communication with the bladder lumen. Bilateral orchiopexy was performed. Complete excision was not attempted because of dense adherence to the posterior bladder wall, attachment of both testes to the lesion, and the risk of injury to the bladder, gonadal structures, or urinary tract in a child with a functionally solitary kidney. The surgical team therefore opened and marsupialized the lesion as a risk-reduction strategy in the setting of uncertain anatomy. An intra-abdominal drain was placed, and a Foley catheter was left *in situ*.

A cyst-wall specimen measuring approximately 2.5 × 1.5 × 0.2 cm was submitted for histopathological examination. Histopathology reported benign intra-abdominal cystic tissue with features interpreted as consistent with mesenteric cyst, without evidence of malignancy. No immunohistochemistry was performed, and the report did not provide detailed epithelial characterization or describe urothelium, transitional epithelium, lymphatic lining, endothelial markers, or mesothelial markers. Therefore, the pathology was considered supportive of mesenteric cyst morphology but insufficient to establish the lesion's complete anatomic origin.

The postoperative course was complicated by fever, leukocytosis, and elevated inflammatory markers. On 20 May 2025, white blood cell count was 18.4 × 10⁹/L. On 23 May 2025, C-reactive protein was 69.9 mg/L. Postoperative culture documentation reported *Klebsiella* sensitive to meropenem; however, the exact culture source, collection method, and colony count were unavailable. The child improved clinically after targeted therapy. After discharge, the intra-abdominal drain was accidentally removed at home. Five days later, the mother reported passage of urine from the abdominal tract during straining, raising concern for a suspected postoperative urinary drainage tract or vesicocutaneous tract. No contemporaneous cystogram, contrast study through the tract, methylene blue test, biochemical analysis of drainage fluid, or operative re-exploration was documented. Follow-up ultrasound on 8 October 2025 demonstrated an empty urinary bladder and a small supravesical soft-tissue-like structure connected to the skin by a tract measuring approximately 1.2 cm in diameter and 3 cm in length. The right kidney was not seen. The left kidney was described as normal in size, shape, echotexture, and position, with normal cortical thickness and no hydronephrosis. Because the reported urine passage occurred after cyst marsupialization and accidental drain removal, the tract was interpreted clinically as more likely postoperative, possibly related to the marsupialized bladder-adherent lesion and/or the drain pathway. However, because preoperative or intraoperative cystourethroscopy was not performed and no early contrast evaluation of the tract was available, a missed pre-existing occult communication cannot be excluded with certainty.

The patient subsequently underwent cystoscopic evaluation on 25 November 2025. According to the treating team, postoperative cystoscopy reportedly excluded posterior urethral valves, bladder duplication, bladder diverticulum, ectopic ureteric opening, and an internal bladder fistulous opening. However, the detailed postoperative cystoscopy report was unavailable; therefore, these findings were interpreted cautiously. Long-term follow-up after this evaluation was incomplete, and the final status of the supravesical tract remains unknown.

## 3 Discussion

This case illustrates the diagnostic difficulty of a posterior bladder-associated pelvic cystic lesion in a male infant with complex urinary tract anatomy. Although the limited cyst-wall specimen showed histologic features interpreted as consistent with mesenteric cyst, the lesion's posterior bladder location, severe ipsilateral kidney abnormality, operative adherence to the posterior bladder wall, and suspected postoperative urinary drainage tract restricted definitive classification. When a cystic lesion lies deep in the male pelvis and closely abuts the bladder, its radiological and operative appearance may overlap with genitourinary anomalies (6–8).

The differential diagnosis in this case included Zinner-spectrum malformation, seminal vesicle cyst, ectopic ureteral insertion, bladder duplication, bladder diverticulum, and mesenteric cyst. Zinner syndrome is classically defined by seminal vesicle cyst, ipsilateral ejaculatory duct obstruction, and ipsilateral kidney agenesis or severe abnormality (7, 8). Although rare in children, Zinner-spectrum variants are relevant in male patients with posterior bladder-associated pelvic cystic lesions and ipsilateral upper urinary tract anomalies (7, 8). In the present case, the combination of male sex, posterior bladder-associated pelvic cystic lesion, and severe right kidney abnormality made Zinner-spectrum malformation and ectopic ureter-related pathology important considerations. Bladder duplication and bladder diverticulum were also considered because the lesion resembled a bladder-associated structure radiologically and intraoperatively (Table 1).

**Table 1:** Differential diagnosis of the posterior bladder-associated pelvic cystic lesion.

Differential diagnosis	Why it was considered	Findings against or limiting certainty
Zinner-spectrum malformation	Male patient; posterior bladder-associated pelvic cystic lesion; severe ipsilateral right kidney abnormality.	Seminal vesicle origin and ejaculatory duct obstruction were not documented; detailed postoperative cystoscopy report was unavailable; infant age limited clinical characterization of seminal vesicle and ejaculatory duct anatomy.
Seminal vesicle cyst	Posterior bladder-associated location in a male patient.	Direct seminal vesicle origin was not demonstrated on available imaging or operative documentation; detailed postoperative cystoscopy report was unavailable.
Ectopic ureteral insertion	Severe ipsilateral kidney abnormality and posterior bladder-associated pelvic cystic lesion made ectopic ureter-related pathology clinically relevant.	Ectopic ureteric opening was reportedly excluded by postoperative cystoscopy, but the detailed report was unavailable for independent verification; no preoperative or intraoperative cystourethroscopy was performed.
Bladder duplication	Lesion resembled a bladder-associated cystic structure radiologically and intraoperatively.	Postoperative cystoscopy reportedly excluded bladder duplication, but the detailed report was unavailable; no endoluminal confirmation was performed intraoperatively.
Bladder diverticulum	Posterior bladder-associated location and suspected postoperative urinary drainage made diverticular pathology relevant.	No diverticular neck was documented; postoperative cystoscopy reportedly excluded diverticulum, but the detailed report was unavailable.
Mesenteric cyst	Limited cyst-wall histology showed benign cystic tissue with features interpreted as consistent with mesenteric cyst.	Posterior bladder adherence, severe ipsilateral kidney abnormality, limited sampling, absence of immunohistochemistry, lack of detailed epithelial characterization, and suspected postoperative urinary drainage prevent definitive classification.

In this patient, the available records did not document seminal vesicle origin, ejaculatory duct obstruction, ectopic ureteric insertion, or a definite pre-existing bladder lumen communication. Postoperative cystoscopy was reportedly negative for posterior urethral valves, bladder duplication, bladder diverticulum, ectopic ureteric opening, and internal bladder fistula, but the detailed report was unavailable for independent verification. Taken together, the available findings support classification as a posterior bladder-associated pelvic cystic lesion with histologic features consistent with mesenteric cyst, although definitive anatomical confirmation of a mesenteric cyst or communicating lesion was not available. The pathology result is supportive but not definitive. The specimen submitted was a limited cyst-wall fragment, and no immunohistochemistry or detailed epithelial characterization was performed. The report did not describe urothelium, transitional epithelium, lymphatic lining, endothelial markers, or mesothelial markers. Although the pathology report did not describe urothelium or transitional epithelium in the sampled tissue, this absence in a limited report cannot definitively exclude a genitourinary-associated lesion elsewhere in the cyst wall. Limited sampling therefore cannot characterize the entire lesion or its full anatomical relationship to the bladder. The pathological findings therefore support mesenteric cyst morphology but do not independently establish the complete anatomical origin of the lesion.

The operative decision also requires cautious interpretation. Complete excision is generally preferred for mesenteric cysts when safely feasible because incomplete treatment may increase the risk of recurrence or persistent drainage (1, 3, 4). In this case, however, the lesion was densely adherent to the posterior bladder wall, and both testes were attached to its posterior aspect. In a child with a functionally solitary kidney and urinary tract vulnerability, aggressive complete excision could have risked bladder injury, gonadal injury, ureteral injury, or worsening urinary complications. Marsupialization was therefore selected as a risk-reduction strategy in the setting of uncertain anatomy, rather than as a standard definitive approach for an anatomically confirmed mesenteric cyst.

The postoperative supravesical tract further illustrates the complexity of the case. The mother reported urine passage from the abdominal tract after accidental drain removal, and ultrasound later demonstrated a supravesical structure connected to the skin by a tract. This finding is best described as a suspected postoperative urinary drainage tract or vesicocutaneous tract. A postoperative explanation is plausible because the symptom appeared after marsupialization and accidental drain removal, potentially involving the marsupialized cavity, bladder-adherent cyst wall, or drain pathway. However, no early cystogram, contrast study through the tract, methylene blue test, biochemical analysis of drainage fluid, or detailed postoperative cystoscopy report was available for independent review. The tract therefore remained an unresolved postoperative finding, without sufficient documentation to distinguish definitively between a postoperative drainage pathway and an occult pre-existing communication. The presence of a functionally solitary left kidney makes this case clinically significant beyond the cyst itself. Children with a congenital functionally solitary kidney or marked functional asymmetry require risk-stratified long-term follow-up because of the risk of hypertension, proteinuria, and kidney function decline (9, 10). In this case, the history of hydroureteronephrosis, culture-positive suspected urinary infection, and complex posterior bladder-associated pelvic cystic anatomy supports the need for long-term pediatric urology and nephrology follow-up.

The clinical timeline also reflected real-world constraints in staged pediatric surgical evaluation. The diagnostic and operative course was affected by the need for neonatal stabilization, infection treatment, serial imaging, functional kidney assessment, CT characterization, diagnostic uncertainty, family-related delay, and operative scheduling constraints in a resource-limited public healthcare system. These contextual factors help explain the sequence of care but do not remove the diagnostic limitations created by delayed and incomplete anatomical evaluation.

This case has several limitations that directly affect diagnostic certainty. First, preoperative or intraoperative cystourethroscopy was not performed, despite an initial micturating cystourethrogram that could not definitively exclude posterior urethral valves and despite the posterior bladder-associated location of the lesion. Second, although postoperative cystoscopy reportedly excluded posterior urethral valves, bladder duplication, bladder diverticulum, ectopic ureteric opening, and internal bladder fistula, the detailed postoperative cystoscopy report was unavailable for independent verification. Third, the pathology diagnosis was based on limited cyst-wall sampling without immunohistochemistry or detailed epithelial characterization. Fourth, VCUG images, intraoperative photographs or surgical video, pathology images, postoperative ultrasound images of the tract, and additional postoperative tract imaging were unavailable beyond the reviewed reports and CT images. Fifth, the urine collection method, colony count, and complete pyuria documentation for the early culture-positive suspected urinary infection were unavailable, and the exact source and colony count of the postoperative *Klebsiella* culture were also unavailable. Finally, long-term follow-up was incomplete; therefore, the final status of the supravesical tract, recurrence risk, hydronephrosis course, and kidney function trajectory remains unknown. These limitations constrain the final interpretation of the lesion and support the more cautious designation of a posterior bladder-associated pelvic cystic lesion with histologic features consistent with mesenteric cyst.

## 4 Conclusion

Posterior bladder-associated pelvic cystic lesions in male infants may create substantial diagnostic difficulty, particularly when congenital urinary tract anomalies and a functionally solitary kidney are present. This case describes a pelvic cystic lesion with histologic features consistent with mesenteric cyst in which definitive classification was constrained by its posterior bladder location, dense adherence to the posterior bladder wall, severe ipsilateral kidney abnormality, limited cyst-wall sampling, absence of immunohistochemistry, lack of preoperative or intraoperative cystourethroscopy, unavailable detailed postoperative cystoscopy documentation, and incomplete long-term follow-up. The case emphasizes the need for broad differential diagnosis, including Zinner-spectrum malformation, seminal vesicle cyst, ectopic ureteral insertion, bladder duplication, and bladder diverticulum. When complete excision is unsafe because of adherence to critical structures, marsupialization may be selected as a risk-reduction strategy, with attention to possible drainage or recurrence risk and the need for long-term pediatric urology and nephrology follow-up.

## Data Availability

The original contributions presented in the study are included in the article/Supplementary Material, further inquiries can be directed to the corresponding author.
